# Advances in the study of apathy related to cerebral small vessel disease

**DOI:** 10.3389/fneur.2025.1513574

**Published:** 2025-02-12

**Authors:** Shuo-han Bu, Xin-zhu Hu, Zhe Su, Li-tao Li

**Affiliations:** ^1^Department of Neurology, Hebei North College, Zhangjiakou, China; ^2^Department of Neurology, Hebei General Hospital, Shijiazhuang, China; ^3^Department of Neurology, Hebei Medical University, Shijiazhuang, China; ^4^Hebei Provincial Key Laboratory of Cerebral Networks and Cognitive Disorders, Hebei General Hospital, Shijiazhuang, China

**Keywords:** cerebral small vessel disease, apathy, pathogenesis, biomarker, treatment

## Abstract

Cerebral small vessel disease (CSVD) is a complex clinical-imaging pathological syndrome caused by small vessel lesions in the brain, which is characterized by aging-related, insidious onset and slow progression. Apathy is a key component of the common neuropsychiatric symptoms among CSVD patients, severely affecting their daily lives and social functioning. Moreover, there are fewer studies on CSVD-related apathy, and greater attention should be paid to this condition in clinical practice. This article describes the latest research advances in the concept, epidemiological features, pathogenesis, assessment and diagnosis, imaging and biomarkers, and treatment of CSVD-related apathy, aiming to serve as a reference for the clinical diagnosis and prevention of CSVD-related apathy.

## 1 Introduction

Cerebral small vessel disease (CSVD) is linked to a series of clinical, imaging, and pathological syndromes caused by various etiologies affecting small arteries and their distal branches of micro arterioles, capillaries, microvessels, and small veins in the brain (1). CSVD is a type of cerebrovascular disease related to increasing age, and characterized by insidious onset, and slow progression. Imaging markers of CSVD include recent small subcortical infarct (RSSI), lacune of presumed vascular origin, white matter hyperintensity of presumed vascular origin (WMH), perivascular space (PVS), cerebral microbleed (CMB), cortical superficial siderosis (cSS), brain atrophy, cortical cerebral microinfarct (CMI), and Incidental DWI-positive lesion (2). The clinical manifestations of CSVD are highly heterogeneous and include symptoms such as cognitive dysfunction, gait disturbance, affective disorder, and diaphoresis.

Apathy is one of the common neuropsychiatric symptoms in CSVD and has been a significant research topic in recent years (3). Zhao et al. (4) reported that the prevalence of apathy in Alzheimer’s Disease (AD) patients in China was 37. 33% and there was a correlation between apathy and CSVD imaging markers through a cross-sectional study of 150 CSVD patients. Li et al. (5) explored the relationship between CSVD-related apathy, cognitive function, and changes in deep gray matter structures by segmenting the deep gray matter structure of the cerebral hemispheres in CSVD patients using the FIRST tool. Their studies demonstrated that apathy was associated with a smaller volume and altered shape of the striatum, identifying it as an independent factor contributing to cognitive impairment in CSVD patients. However, the above studies have not confirmed that changes in deep gray matter structures mediate apathy and cognitive impairment. This article will discuss recent studies on the concept, epidemiological characteristics, pathogenesis, assessment, imaging and biomarkers, and treatment of CSVD-related apathy, thereby providing a reference for future clinical diagnosis and treatment of CSVD-related apathy.

## 2 Concept and epidemiology of CSVD-related apathy

Apathy, as a behavioral syndrome, is now widely accepted as a decrease in goal-directed activities across cognitive, behavioral, affective, or social domains of a patient’s life (6). Furthermore, related studies have shown that it is associated with cognitive function and overall health status (7). It is widespread in all types of neuropsychiatric disorders, with prevalence rates ranging from 24 to 85% in AD, 43–89% in vascular dementia (8), 20–92% in progressive supranuclear palsy (9), and 10.7–44.8% in mild cognitive impairment (10). Apathy is increasingly recognized as an early clinical manifestation in patients with CSVD and plays a significant role in cognitive function changes and dementia prediction (11). Wang et al. (12) utilized the apathy evaluation scale-clinical (AES-C) to assess apathy in CSVD patients and healthy controls. Their findings revealed that CSVD patients had higher AES-C scores and a greater incidence of apathy compared to the control group. Similarly, Cai et al. (13) found in a cross-sectional study that the prevalence of apathy among Chinese CSVD patients reached 37.50%. Additionally, other studies have shown that apathy affects more than one-third of hospitalized older adults with CSVD (4). Xia et al. (14) reported that the diagnostic rate of apathy syndrome in elderly patients with sporadic CSVD was 34.33%, with the most significant reductions observed in cognitive and behavioral activities, followed by social activities. However, the study’s relatively small sample size limits the generalizability of these results, highlighting the need for future research. In summary, these findings indicate that apathy is relatively common in CSVD patients and is closely related to impairments in daily functioning and cognitive ability. With the aging population, the incidence of CSVD-related apathy is on the rise, which demonstrates the seriousness of the issue of CSVD-related apathy.

## 3 Pathogenesis of CSVD-related apathy

Research on the pathogenesis of CSVD-related apathy is still in its early stages. Most studies have focused on the pathogenesis of apathy in neurodegenerative diseases and healthy populations, with the underlying mechanisms remaining poorly understood. Some studies proposed the hypothesis of dysfunctional cortico-subcortical circuits, suggesting that reduced microstructural integrity of white matter is closely related to apathy, particularly in the limbic association bundles such as the anterior cingulate, fornix, and leptomeningeal bundles (15). Additional literature indicates that various regions of the prefrontal cortex are associated with different dimensions of apathy. For example, the dorsolateral prefrontal cortex is related to the generation of cognitive planning or action goals, the dorsomedial prefrontal cortex is associated with self-initiated actions, and the orbitofrontal cortex is related to emotional evaluation (16). Furthermore, Tay et al. (3) proposed a brain network-based model conceptualizing apathy as a result of damage to goal-directed behavior (GDB)-related networks. This model correlates focal or diffuse lesions of CSVD with neurobiological changes. It suggests that focal lesions, such as lacunar infarcts (LI) occurring at key nodes within brain networks, lead to a decrease in the efficiency of these nodes, impairing the function of the entire network. Similarly, diffuse lesions such as WMH lead to extensive loss of white matter fiber connectivity, resulting in inefficient brain network connectivity. Other studies have shown that poorer structural and functional coupling of brain networks, along with lower cognitive function, correlates with the severity of apathy (17, 18). These neurobiological changes not only negatively impact patient’s daily lives but also have a long-lasting negative effect on their cognitive functioning. In addition, Saleh et al. (19) investigated the effect of altered responses to reward and effort on apathy by using Bayesian drift-diffusion modeling. They found that apathetic patients had a poor response to low-level rewards, and more apathetic patients accepted fewer proposals under high effort. This suggests that the efficiency of the reward network, which is primarily governed by dopamine-projecting fibers, is negatively correlated with apathy. In recent years, some studies have proposed the vascular apathy hypothesis, which suggests that CSVD is the primary causative factor of apathy and is the only cause of apathy. This hypothesis is supported by the findings that apathy is associated with the traditional vascular risk factors for CSVD, such as hypertension and cardiovascular diseases. However, the relationship between CSVD and apathy still lacks robust evidence and remains somewhat controversial (20). In summary, dysfunction of cortico-subcortical circuits, impairment of brain networks, and deficits in effort-based reward decision-making caused by impaired dopamine transmission may represent the main pathogenesis mechanisms underlying CSVD-related apathy.

## 4 Assessment of CSVD-related apathy

The assessment of CSVD-related apathy primarily consists of scale-based assessment and objective assessment. Currently, no assessment tool is universally recognized as the “gold standard.” Scales for the assessment of apathy focus on assessing an individual’s performance of apathy in affective, cognitive, and behavioral domains. These scales have been widely applied in various studies to assess apathy symptoms in AD, mild cognitive impairment, and other neurodegenerative disorders. Common scales include the Apathy Evaluation Scale (AES), Neuropsychiatric Inventory (NPI), Geriatric Depression Scale (GDS), Frontal Systems Behavior Scale (FrSBe), Apathy Inventory (AI), and Caregiver Burden Scale (CBS), etc. (4, 21, 22). MARIN proposed the AES, which consists of 18 items, including self-reported (AES-S), informant-reported (AES-I), and clinician-interviewed (AES-C) dimensions, can assess and quantify the emotional, behavioral, and cognitive aspects of apathy. The total AES score ranges from 0 to 72. Although no clear reference value has been established, the higher the AES score, the greater the severity of apathy (23). Furneri et al. (24) conducted an AES-C assessment on patients with mild cognitive impairment and AD compared to a healthy control group. Their results showed that AES-C was effective for assessing apathy in mild cognitive impairment and AD patient groups. Additionally, the NPI apathy measure consists of 1 screening question and 8 assessment questions, scored on a scale of 0 to 12. A score of 3 to 4 or higher is generally defined as apathy, with higher scores indicating greater severity. While the GDS primarily assesses depression in older adults, some studies have found that the GDS is also suitable for evaluating apathy. It allows for the analysis of both conditions, which can help better differentiate between apathy and depression and provide more accurate treatment plans for patients. Therefore, different assessment scales can be selected based on specific clinical situations, or multiple scales may be used in combination for a more comprehensive evaluation. Some scholars have argued that despite the widespread use of apathy assessment scales, there is a lack of an objective basis for determining their cut-off values. In addition, there are some variabilities in how raters are being trained to administer and score the instrument in different clinical trials, which may affect the accuracy of the apathy diagnosis (25).

Cai et al. (13) conducted a cross-sectional study of patients with CSVD by using a somatic motion analyzer. They found that the daily sagittal amplitude was lower in CSVD patients with apathy compared to those without apathy. Additionally, the diurnal daily sagittal amplitude and the total bedtime of CSVD patients were negatively correlated with the severity of apathy. This suggests that the somatic motion analyzer is an objective tool for measuring sleep quality and a promising technique for assessing CSVD-related apathy. Consequently, the somatic motion analyzer is a noninvasive and simple tool, and current studies highlight its potential for detecting CSVD-related apathy, thereby offering significant assistance for early intervention in apathy. Furthermore, information and communication technologies, such as infrared sensors and, remote monitoring (26), provide potentially objective methods for the diagnosis of apathy and allow for a more accurate assessment of it. In summary, the accuracy of apathy assessment can be improved through the combined use of scale-based assessment and advanced information and communication technology. Early identification and intervention in apathy are important for improving patients’ quality of life and prognosis.

## 5 Imaging and biomarkers of CSVD-related apathy

### 5.1 Imaging markers of CSVD-related apathy

Brain magnetic resonance imaging (MRI) is the most commonly used method for diagnosing CSVD, enabling the visual assessment of CSVD-related apathy by detecting hemorrhagic and ischemic brain parenchymal lesions in CSVD (27). Kleber et al. (28) measured the relationship between WMH volume and CSVD-related apathy using automated brain MRI segmentation software. They found that patients with CSVD and apathy exhibited a larger WMH volume, suggesting that disruption of extensive white matter structures plays a key role in the development of apathy. Research indicates that brain network disruption underlies the relationship between CSVD imaging markers and apathy, with apathy being related to impaired network connectivity in premotor and cingulate regions (29). Furthermore, studies have shown that patients with CSVD and apathy have higher Fazekas scores compared to non-apathetic patients (30), which is consistent with previous findings. Clancy et al. (31) conducted an in-depth analysis of apathetic subtypes (executive, affective, and initiating apathy), demonstrating that executive and affective apathy were more strongly associated with WMH than initiating apathy. As research progresses, the association between apathy and white matter regions of the brain responsible for emotional processing has been gradually recognized. The severity of apathy is positively correlated with the severity of WMH (13, 14). These findings may provide potential therapeutic targets, though further validation is needed in future studies. Another major imaging marker of CSVD-related apathy is lacunar. TAY et al. (29) used pathway analysis to demonstrate a correlation between lacunar cerebral infraction and apathy, consistent with previous studies. Douven et al. (32) found more severe brain atrophy and smaller right hippocampal volumes in patients with post-stroke apathy in a prospective cohort study, suggesting a correlation between brain atrophy and apathy. However, the relationship between apathy and other markers of CSVD is currently controversial and requires further research.

In recent years, an increasing number of studies have shown that the severity of apathy is related to cortical thickness. In a study on frontotemporal dementia with apathy, it was found that the severity of apathy was significantly associated with cortical thinning in the lateral regions of the right frontal, temporal and parietal lobes (33). Furthermore, a clinically based longitudinal memory study Matuskova et al. (34) showed that the formation of apathy was strongly related to lower hippocampal volume. In conclusion, WMH, lacunar cerebral infarction, cerebral atrophy, changes in cortical thickness and hippocampal volume are important imaging markers of CSVD-related apathy, which are helpful for diagnosis and prognostic assessment.

### 5.2 Apathy-related biomarkers

Studies on biomarkers of apathy have been a hot research topic in recent years, although most have focused on populations with neurodegenerative diseases. Biomarkers of CSVD-related apathy remain relatively understudied and will need refinement in future research. Sun et al. (35) found that frontal Amyloid *β*(Aβ) deposition was related to the risk of apathy development by cerebrospinal fluid immunoassay in AD patients. Furthermore, apathy has been associated with a higher brain Aβ burden, indicating a bidirectional relationship between apathy syndrome and Aβ pathology. The study also revealed that the impact of apathy severity on cognition and quality of life was related to prefrontal area Aβ. However, the study’s small sample size and controversial results highlight the need for further validation with larger multidimensional analyses. In an animal model of AD, investigators found that Aβ triggered intermittent aberrant excitatory neuronal activity in the cerebral cortex and hippocampus, leading to significant remodeling of inhibitory circuits and increased inhibition of granule cells. This process was shown to contribute to the development of apathy (36). However, it is important to note that this study was conducted on AD animal models, with several limitations, which require further validation in human studies. Matteo et al. (37) analyzed the correlation between cerebrospinal fluid tau protein levels and neuropsychiatric symptoms. Their results indicated that apathy scores were positively correlated with cerebrospinal fluid total tau protein (t-tau) and phosphorylated tau protein (p-tau) levels. Other studies have identified tau proteins in the orbitofrontal cortex as potential contributors to focal neurotoxicity in the orbitofrontal cortex, which in turn disrupts the orbitofrontal cortex-leptomeningeal bundle network, leading to the emergence and progression of apathy in AD patients. Future studies should cover a broader range of brain regions to further investigate their relationship with apathy (38). In addition, studies have indicated a significant interaction between cerebral microbleeds and cerebrospinal levels of total tau (t-tau) and Aβ42 in apathy, suggesting that these biomarkers and imaging markers are synergistic to some extent (39). Additionally, some studies suggested that brain-derived neurotrophic factors and tumor necrosis factor-alpha were related to apathy, but the studies are still scarce and need to be verified by further research. Currently, biomarker studies of apathy are focused on neurodegenerative disease populations, and markers of CSVD-related apathy are relatively understudied. Therefore, further studies are needed to improve this area in the future. In summary, studies on neurodegenerative diseases suggest that imaging and biomarker studies can provide a deeper understanding of the pathogenesis of CSVD-related apathy and represent a reference for clinical diagnosis and treatment.

## 6 Treatment of CSVD-related apathy

In current clinical practice, trials of pharmacologic treatments for apathy symptoms in patients with neurodegenerative diseases are underway, which offer new options for the treatment of apathy. At present, the treatment of apathy mainly includes two types: pharmacological therapy and non-pharmacological therapy. The study suggested that dysfunction of cortico-subcortical circuits was the primary mechanism for the development of apathy (15). Christopher et al. (16) found that methylphenidate improved apathy by enhancing norepinephrine and dopamine activity in the prefrontal-striatal-thalamocortical circuit. A randomized controlled trial by Chittaranjan et al. (40) showed that while methylphenidate reduced the severity of apathy, it did not improve daily activities or quality of life. Therefore, this has led to some controversy regarding the clinical significance of methylphenidate, and more in-depth research is needed. Additionally, some studies have demonstrated the clinical effectiveness of dopamine receptor agonists in treating apathy in Parkinson’s disease, while cholinesterase inhibitors also play a supportive role in managing apathy (41). Selective 5-hydroxytryptamine reuptake inhibitor (SSRI) has been associated with an increased incidence of apathy, which is consistent with previous findings (42). Ketamine has been proved reversing motivation-related deficits in reward processing, but it has not yet been tested for the treatment of apathy and could be intensively studied as a potential treatment in the future (43). Additionally, non-pharmacological therapies include exercise therapy, music therapy, transcranial direct current stimulation, and repeated Transcranial Magnetic Stimulation (rTMS), etc. (44, 45). Exercise therapy improves mood by increasing endorphin release and enhancing social activity. Music therapy helps improve the living environment and promote emotional expression and regulation (44). Lisoni et al. (45) conducted a randomized controlled trial and found that bilateral prefrontal transcranial direct current stimulation (tDCS) could effectively improve apathy in schizophrenic patients. Lefaucheur et al. (46) found that rTMS could regulate neuronal electrical activity and change synaptic plasticity, and thus produce long-lasting potential effects, inducing reconnection of brain networks after interruption. There is no approved pharmacologic treatment for apathy, and thus the treatment of CSVD-related apathy faces significant challenges.

## 7 Prospects

Apathy is a specific manifestation of CSVD and plays a significant role in determining the quality of life of CSVD patients. However, at present, the mechanism of CSVD-related apathy remains largely speculative. Future studies should focus on exploring the specific pathogenesis mechanisms of CSVD-related apathy. Additionally, the development of new diagnostic tools and biomarkers to more accurately identify and quantify CSVD-related apathy represents an important direction for future research. Finally, large-scale international collaborative studies to collect more clinical information on CSVD-related apathy would enhance our understanding of the condition and provide a robust scientific basis for developing future therapeutic strategies.
